# Fruit Food

**Published:** 1879-08

**Authors:** 


Fruit Food.—This is a laxative mixture of fruit and oats and coarse wheat, made palatable with honey. It has all the scratching, scouring power of coarse foods, and thus induces somewhat active movements of the bowels. It acts a good deal like Graham bread, only more so. It is probably better than pills or other purgatives, and is certainly pleasanter to take. The Health Food Co. look upon all such things as matters to be avoided, because it believes that the world needs feeding, not physicing, and that it will have good digestion when fed just right. Still, for those who can't get proper food, and who, therefore, suffer from torpid bowels, the Fruit Food comes in as a ready aid and comforter.

